# Identification of TUBB4A as a Prognostic Biomarker of Melanoma by Transcriptomic Data and *In Vitro* Experiments

**DOI:** 10.1177/15330338231184842

**Published:** 2023-07-12

**Authors:** Jiaqi Zhang, Yu Zhang, Jun Liu, Judong Luo, Yifei Yun, Yan Cao

**Affiliations:** 1 Department of Dermatology, The Affiliated Changzhou No.2 People's Hospital of Nanjing Medical University, Changzhou, Jiangsu Province, China; 2 Department of Radiotherapy, The Affiliated Changzhou No.2 People's Hospital of Nanjing Medical University, Changzhou, Jiangsu Province, China; 3 Department of Radiotherapy, Beijing West Cancer Hospital, Beijing, China; 3 Department of Radiation Oncology, Changzhou Fourth People's Hospital, Changzhou, Jiangsu Province, China

**Keywords:** melanoma, TUBB4A, prognostic biomarker, tubulin, differential expression

## Abstract

**Background:** Melanoma is one of the most malignant skin carcinomas with high metastatic potential. Increasing evidence has demonstrated that β-tubulin 4A (TUBB4A) plays a key role in the development and progression of several types of human cancer. However, the potential function of TUBB4A in cutaneous melanoma remains to be determined. **Methods:** We first performed a differential expression analysis based on skin melanoma tissues and normal tissues from Gene Expression Omnibus (GEO) and The Cancer Genome Atlas (TCGA) datasets and then a survival analysis to identify prognostic-related key genes. We further conducted *in vitro* biochemical experiments to verify the functional roles of the key gene TUBB4A. Two small-molecule inhibitors of TUBB4A, Dihydroartemisinin (DHA) and Nocodazole, were used to validate the effect of TUBB4A on the apoptosis and cell cycle of melanoma cells. **Results:** We found that TUBB4A expression was positively correlated to the overall survival (OS) of cutaneous melanoma patients. The coexpressed genes with TUBB4A were enriched in melanoma-related pathways and functions. The experimental results showed that knockdown of TUBB4A inhibited the proliferation and migration of A375 and B16-F10 melanoma cells. Moreover, DHA and Nocodazole promoted the apoptosis of melanoma cells and blocked the melanoma tumor cell cycle in the G2/M stage. **Conclusion:** TUBB4A may be a prognostic biomarker and therapeutic target for melanoma.

## Introduction

Malignant melanoma is the most lethal skin cancer, with an incidence rising rapidly over the past few decades. It is estimated that nearly 1.5 million skin cutaneous melanoma (SKCM) survivors live in the United States, and 99 780 were newly diagnosed with invasive SKCM in 2022. About 40% of melanoma survivors (569 900) are younger than 65 years, and 12% (177 310) are younger than 50 years.^1^ Given its strong invasion and metastasis, novel biomarkers have been explored to realize a more immediate diagnosis and effective treatment of melanoma, such as S100,^2^ HMB-45,^3^ Melan-A,^4^ CSPG4.^5^ Nonaka et al^2^ reported that S100 is the most sensitive marker for melanoma, particularly its subtypes S100A1, S100A6, and S100B. Oncogenic mutants lead to malfunction of driver genes, such as NRAS,^6^ HRAS,^7^ and BRAF.^8^ Potent oncogenes may mutate in benign nevi, such as NRAS in congenital nevi,^6^ HRAS in Spitz nevi,^7^ and BRAF in acquired nevi.^8^ Some tumor suppressor genes lose their functions, such as CDKN2A,^9^ TP53,^10^ and PTEN,^11^ making them potential clinical diagnostic and prognostic markers of melanoma. The deletion, mutation, or silencing of CDKN2A disables p16 in most models of melanoma.^9^ Nevi with histopathological dysplasia show recurrent loss of heterozygosity of CDKN2A and TP53 loci.^12^ Moreover, BRAF activation may cooperate with PTEN loss to drive melanoma development.^11^ Although the prognosis of metastatic melanoma has been greatly improved by targeted and immune therapies, novel biomarkers are still needed to design more efficient diagnostic and therapeutic strategies.

Microtubules, rigid and hollow fibers composed of the cell cytoskeleton, serve to maintain cell shape, cell motility, and cell cycle.^13^ Altered microtubule dynamics is a hallmark of carcinogenesis, and overexpression of β-tubulins is associated with the progression and chemoresistance in different cancers,^14^ such as βIII- and βIV-tubulins in pancreatic tumors.^15^ Haider et al^16^ also reported that aberrant expression of β-tubulins is common in metastatic cancer cells.

TUBB4A is a member of the β-tubulin family and encodes β-tubulin 4A. β-Tubulin is involved in several intracellular processes like mitosis, motility, and transport.^17^ Previous studies have shown GLUT1 and its binding partner TUBB4 as potential targets in glioblastoma multiforme. Silencing TUBB4 inhibits glioblastoma stem cell tumorsphere formation, self-renewal, and proliferation *in vitro*.^18^ The downregulation of βIVa-tubulins in lung cancer cells increases their sensitivity to tubulin-binding agents, such as vinorelbine, vincristine, and paclitaxel.^19^ Ross et al^20^ explored the impact of hypoxia on the proteome and found that TUBB4A differential expression is primarily involved in the structural and binding processes of prostate cancer. Atjanasuppat et al^21^ also reported that ERK signaling mediates the upregulation of βIVa-tubulin genes and confers H460 floating lung cancer cells with resistance against paclitaxel. However, the potential function of TUBB4A in melanoma has not been reported to date.

In this study, we conducted differential expression analysis on two large SKCM cohorts and found that TUBB4A was significantly upregulated in SKCM tissues. A significant correlation between TUBB4A expression and patients’ overall survival (OS) was observed. Moreover, our *in vitro* experiments showed that TUBB4A knockdown reduced the migration and proliferation of melanoma cells. Two drugs in DrugBank, Dihydroartemisinin (DHA) and Nocodazole, were reported to target TUBB4A. Flow cytometry showed that both drugs significantly induced cell apoptosis and G2/M cell cycle arrest. In summary, TUBB4A may serve as a novel therapeutic target for treating melanoma.

## Materials and Methods

### Gene Expressions and Differential Expression Analysis

The gene expression profiles of SKCMs were downloaded from the Gene Expression Omnibus (GEO) (https://www.ncbi.nlm.nih.gov/geo/) and The Cancer Genome Atlas (TCGA) (http://cancergenome.nih.gov/) databases.^22^ From the GEO, we selected human melanoma datasets according to the criteria: sample sizes were larger than 10, and both tumor and nontumor samples were included. As a result, three GEO datasets GSE46517, GSE15605, and GSE3189 were selected, involving 207 tumor samples and 31 normal samples. Details of the three datasets are listed in Table 1. The differential expression analysis was conducted based on an integration of TCGA and GTEx datasets, and differentially expressed genes (DEGs) between melanoma and normal samples were screened as described in our previous study.^23^

**Table 1.** GEO Datasets Enrolled in the Study.

Database	Sample		Platform
	Normal	Melanoma	
GSE46517	8	104	Affymetrix U133A
GSE15605	16	58	Affymetrix U133 Plus 2.0
GSE3189	7	45	Affymetrix U133A

The *Limma* R package was used to conduct differential expression analysis.^24^ The genes with *P*-value < .05 and absolute fold change > 2 were considered DEGs. The R package *ggplot2* was used to draw the volcano plots of DEGs. A Venn diagram was plotted to obtain the overlapping DEGs among the three datasets mentioned above.

### Enrichment, Survival, and Coexpression Analyses

Gene Ontology (GO) and Kyoto Encyclopedia of Genes and Genomes (KEGG) enrichment analyses were performed on the overlapping DEGs using the R package clusterProfiler.^25^ Enriched GO terms in three aspects, including biological process (BP), cellular composition (CC), and molecular function (MF) terms, were obtained.

Survival analysis was conducted on 458 SKCM patients with RNA-seq expression and clinical data downloaded from TCGA. Characteristics of melanoma patients are listed in Table 2. The patients were divided into high- and low-score groups according to the TUBB4A median expression level. The prognostic effect of TUBB4A on OS and disease-free survival (DFS) was estimated using the Kaplan–Meier method, and survival curves were evaluated using the log-rank test.

**Table 2.** Clinical Characteristics of SKCM Patients in TCGA Dataset.

Covariates		TCGA set (*n* = 458)
Age, years (mean ± SD)		57.9 ± 15.5
Gender, no. (%)	Male	174 (37.9)
	Female	284 (62.1)
Stage, no. (%)	I	93 (20.3)
	II	136 (29.7)
	III	170 (37.1)
	IV	22 (4.8)
	Unknown	37 (8.1)
Pathological T, no. (%)	T1	72 (15.7)
	T2	76 (16.6)
	T3	89 (19.5)
	T4	148 (32.3)
	Unknown	73 (15.9)
Pathological N, no. (%)	N0	226 (49.7)
	N1	73 (16.0)
	N2	49 (10.7)
	N3	55 (12.0)
	Unknown	53 (11.6)
Vital status, no. (%)	Alive	249 (54.4)
	Dead	209 (45.6)

SKCM, skin cutaneous melanoma.

Coexpressed genes of TUBB4A were clustered and demonstrated by the heatmap generated by LinkedOmics.^26^ The top 50 genes positively and negatively correlated with TUBB4A were identified by Pearson’s correlations (*P*-value < .05).

### Immunohistochemical Staining

To verify the protein expression level of candidate genes in melanoma tissues, we used the Human Protein Atlas (HPA, https://www.proteinatlas.org/) database to obtain immunohistochemical staining.

### Cell Lines and Cell Culture

Commercially available melanoma cell lines A375 and B16-F10 were purchased from the Shanghai Cell Bank of the Chinese Academy of Sciences (Shanghai, China). A375 and B16-F10 cells were cultured in Dulbecco's modified Eagle's medium (DMEM) containing 10% Fetal bovine serum (FBS) at 37 °C in a humidified incubator with 5% CO_2_.

### Small Interfering RNA Transfection

The specific small interfering RNA (siRNA) was constructed using custom-made siRNA targeting the TUBB4A mRNA region (siTUBB4A sense: GGAGGUUAUCAGUGACGAATT, siTUBB4A antisense: UUCGUCACUGAUAACCUCCTT) and negative control (siNC sense: UUCUUCGAACGUGUCACGUTT, siNC antisense: ACGUGACACGUUCGGAGAATT) (GenePharma, China). Cells were cultured in 6-well plates and transfected with lip3000 (Invitrogen, Carlsbad, CA, USA) following the instructions of the manufacturer. After 48 h of transfection, the cells were harvested for subsequent experiments.

### RNA Isolation and Quantitative Real-Time PCR

A PrimeScript RT reagent kit with gDNA Eraser (Takara, Tokyo, Japan) was used to prepare cDNA. Quantitative real-time PCR (qRT-PCR) was performed by using SYBR Green II Mixture (Takara) according to the manufacturer's protocol; 18S was used for normalization, and the comparative Ct method (ΔΔCt) was used to evaluate mRNA expression. The specific primer pairs were as follows: 18S (internal control gene) primer (forward primer, 5′-GGAGAGGGAGCCTGAGAAACG-3′; reverse primer, 5′-TTACAGGGCCTCGAAAGAGTCC-3′) and TUBB4A primer (forward primer, 5′-CCGGACAACTTCGTGTTTGG-3′; reverse primer, 5′-TCGCGGATCTTACTGATGAGC-3′).

### Cell Proliferation Assay

For the CCK8 assay of cell proliferation, A375 and B16-F10 cells were transfected as previously described. Then, every 800 cells were resuspended in 100 µL of DMEM supplemented with 10% FBS and then added to a 96-well plate. At 24, 48, 72, and 96 h, cell proliferation was investigated using CCK8 (Dojindo Molecular Technologies, USA) according to the manufacturer's instructions. All experiments were performed independently at least three times.

### Wound Healing and Transwell Assay

Cells were seeded in 6-well plates and cultured for 48 h to an approximately 100% confluence. A sterile pipette tip was used to scratch a linear wound, and serum-free DMEM was added for further culturing. Wound healing images were captured at 0, 24, and 48 h using an inverted microscope. For the Transwell assay, 5 × 10^4^ melanoma cells were seeded into the upper well (Corning, USA). The lower chamber was filled with 700 µL of DMEM containing 20% FBS. Transwell chambers were placed in an incubator (37 °C, 5% CO_2_) for 12 h. The cells in the upper chamber were fixed with 4% paraformaldehyde for 15 min, stained with 0.1% crystal violet for 15 min, and counted under an inverted microscope. All experiments were performed independently at least three times.

### Medication and Flow Cytometry

TUBB4A inhibitors were retrieved from DrugBank.^27^ Two small-molecule agents, DHA and Nocodazole, are reported to bind TUBB4A protein. The cells seeded in one 6-well plate were treated with DHA (20 and 40 μM) for 48 h and Nocodazole (0.075 and 0.15 μM) for 24 h, separately.^28,29^ An Annexin V-FITC/PI kit (Becton Dickinson, USA) was used to measure the apoptosis of melanoma cells. All operations were carried out strictly according to the manufacturer's instructions. In brief, the cells were harvested and washed with phosphate buffer saline (PBS), resuspended in 1× binding buffer, and stained with 5 μL of FITC-Annexin and 5 PI for 15 min in the dark. Cell clumps were removed by passing through a cell strainer. The apoptosis of cells was measured by a FACSCalibur flow cytometer (BD Biosciences, USA). The cells in early and late apoptosis were summed, and the total apoptotic rate was calculated.

A single-cell suspension was prepared, and the cells were subsequently fixed with 70% ethanol at 4 °C for 12 h. The fixed cells were then stained with 500 μL working solution (0.02% RNase solution, 0.02% PI solution, and 0.1% assay buffer) at 37 °C for 30 min and 4 °C for 30 min. Cell clumps were removed by passing through a cell strainer. Data were acquired using BD Accuri Cytometry (BD BioSciences, USA). All experiments were performed independently at least three times.

### Statistical Analysis

Statistical analyses were performed using SPSS 23.0 software (IBM, Chicago, IL, USA) and GraphPad Prism 8.0 (GraphPad Software, La Jolla, CA, USA). Student's *t*-test was used to analyze the difference between the two groups. One-way analysis of variance (ANOVA) was used for the comparison among three or more groups. *P*-values less than .05*, less than .01**, and less than .001*** were considered statistically significant. Each cytological experiment was performed independently at least three times.

## Results

### Differentially Expressed Genes Related to Melanoma

**Figure 1.**
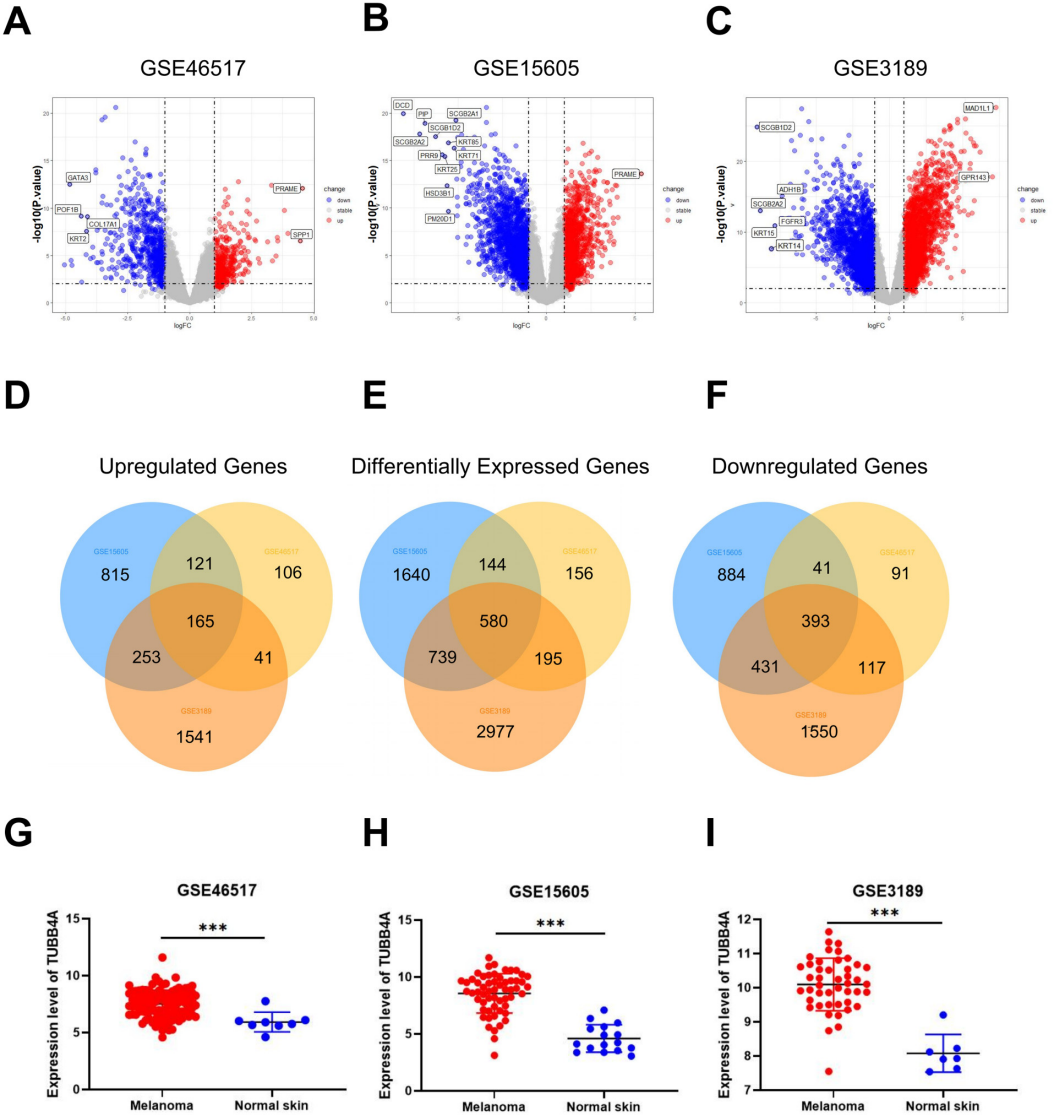
Differentially expressed genes (DEGs) between normal and melanoma tissues (fold change > 2, *P*-value < .05). (A-C) Volcano plots of the *P*-value as a function of weighted fold change for DEGs; red dots represent significantly upregulated expressed genes, and blue dots represent significantly downregulated expressed genes. (D-F) Overlapping DEGs in datasets. Every circle corresponds to a dataset. The numbers of DEGs in each overlapped area are marked in relevant position. DEGs within three datasets are regarded as credible in each Venn diagram. (G-I) mRNA expression of TUBB4A in melanoma and normal tissues in the GEO dataset. *P *<* .05; **P < .01; ***P < .001. The data were analyzed using Student's *t*-test.

Differential expression analyses were conducted on three GEO datasets independently, and the volcano plots of DEGs (*P*-value < .05, absolute fold change > 2) were displayed in Figure 1A to C. Subsequently, 580 overlapped DEGs in three SKCM datasets were filtered out and illustrated in the Venn diagram, including 165 upregulated and 393 downregulated (Figure 1D to F). Moreover, TUBB4A overexpression was found in melanoma tissues in all three datasets (Figure 1G to I).

**Figure 2.**
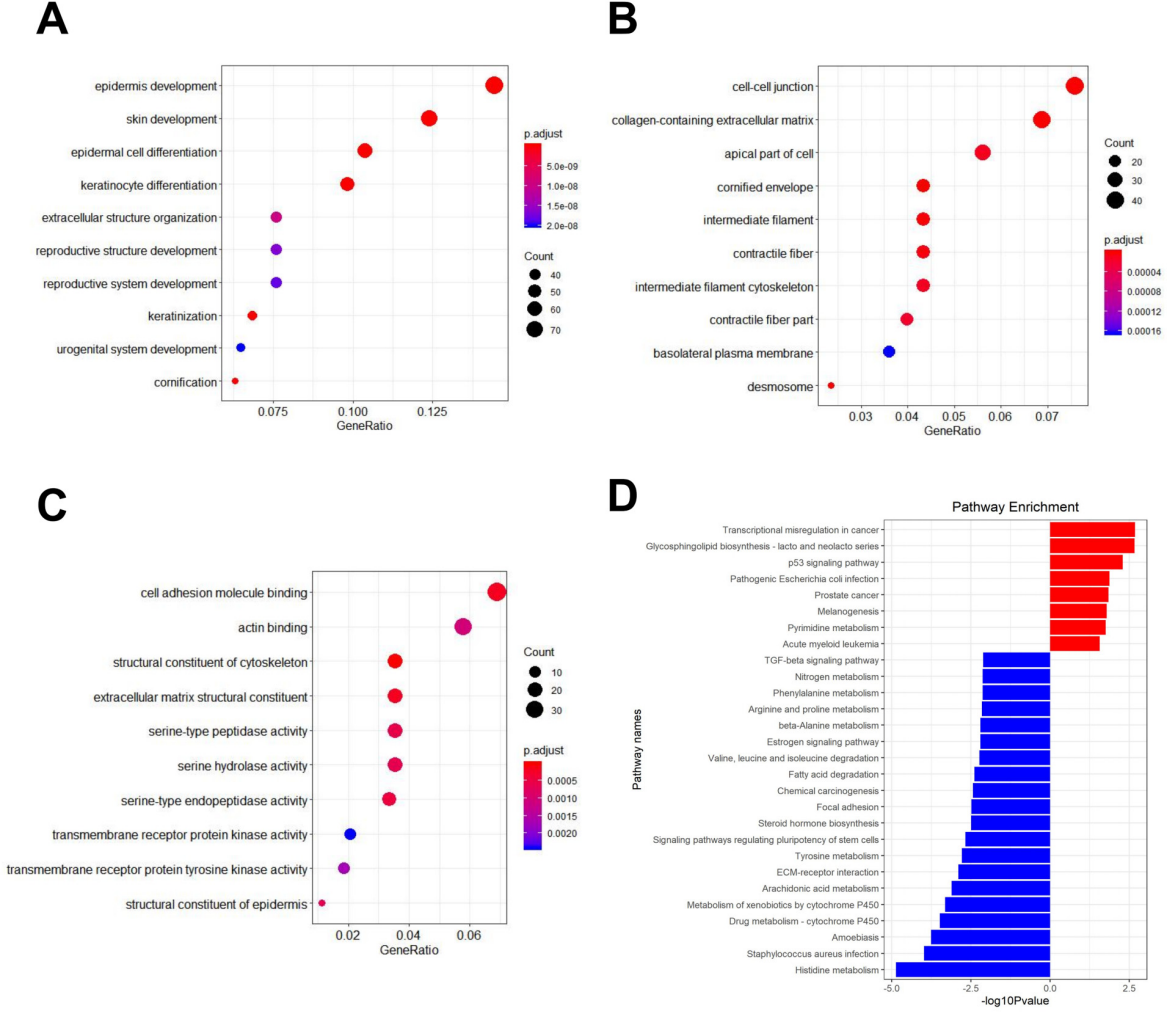
GO and KEGG pathway enrichment analyses. (A-C) GO BPs, CCs, and MFs of DEGs. (D) KEGG pathway analysis of DEGs.

Abbreviations: GO, Gene Ontology; KEGG, Kyoto Encyclopedia of Genes and Genomes; BPs, biological processes; CCs, cellular compositions; MFs, molecular functions; DEGs, differentially expressed genes.

GO analysis showed that the DEGs were significantly enriched in skin- and epidermis-related BP terms, such as skin and epidermis development, epidermal cell and keratinocyte differentiation, and extracellular structure organization. As for CC terms, these DEGs were significantly enriched in cell–cell junction, collagen-containing extracellular matrix, apical part of cell, cornified envelope, and intermediate filament. As for MF terms, the DEGs were significantly enriched in cell adhesion molecular binding, actin binding, structure constituent of cytoskeleton, extracellular matrix structural constituent, and serine-type peptidase activity (Figure 2A to C).

KEGG enrichment analysis showed that the top 25 significantly enriched pathways were all related to cancerogenesis (Figure 2D). Upregulated genes were mainly enriched in the pathways related to transcription misregulation in cancer, glycosphingolipid biosynthesis-lacto and neolacto series, p53 signaling pathway, and melanogenesis. Downregulated genes were significantly enriched in histidine metabolism, *Staphylococcus aureus* infection, amebiasis, drug metabolism, and metabolism of xenobiotics by cytochrome P450.

It has been reported that melanin is partially attributed to the secretion of ɑ-melanocyte-stimulating hormone (ɑ-MSH) induced by keratinocytes, while the ultraviolet (UV)-induced secretion of ɑ-MSH in the skin is directly regulated by p53.^30^ Key signal molecule p53 is activated in response to DNA damage to promote the proliferation of melanoma and thyroid cancer cells. Moreover, it also mediates cell cycle arrest, apoptosis, and senescence.^31^

### TUBB4A Overexpression Is Correlated to Prognosis in Melanoma

The differential expression analysis revealed that 1485 genes were differentially expressed between tumor and normal samples, including 514 upregulated and 972 downregulated. The Kaplan–Meier analysis showed that five upregulated genes were significantly related to both the OS and DFS of melanoma patients, including TUBB4A, PSEN2, SLC45A2, QPRT, and TRPV2 (Table 3).

**Table 3.** Genes Significantly Related to the Prognosis of Patients with Melanoma.

Gene symbol	*P-*value	
	OS	DFS
TUBB4A	.000039	.013
PSEN2	.0017	.028
SLC45A2	.002	.048
QPRT	.030	.016
TRPV2	.039	.016

OS, overall survival; DFS, disease-free survival.

An extensive literature review was conducted to verify the genes associated with the survival of melanoma patients. SLC45A2 is involved in melanosome maturation and pigmentation and associated with the risk of cutaneous malignant melanoma.^32,33^ The upregulation of PSEN2 increases human melanoma aggressiveness and worsens prognosis, and it is also the target of MYC.^34^ According to a previous study,^35^ TRPV2 exhibited ectopic distribution in both melanocytes and melanoma cells. Moreover, activation of TRPV2 could lead to the decline of the viability of melanoma A2058 and A375 cells.^35^

**Figure 3.**
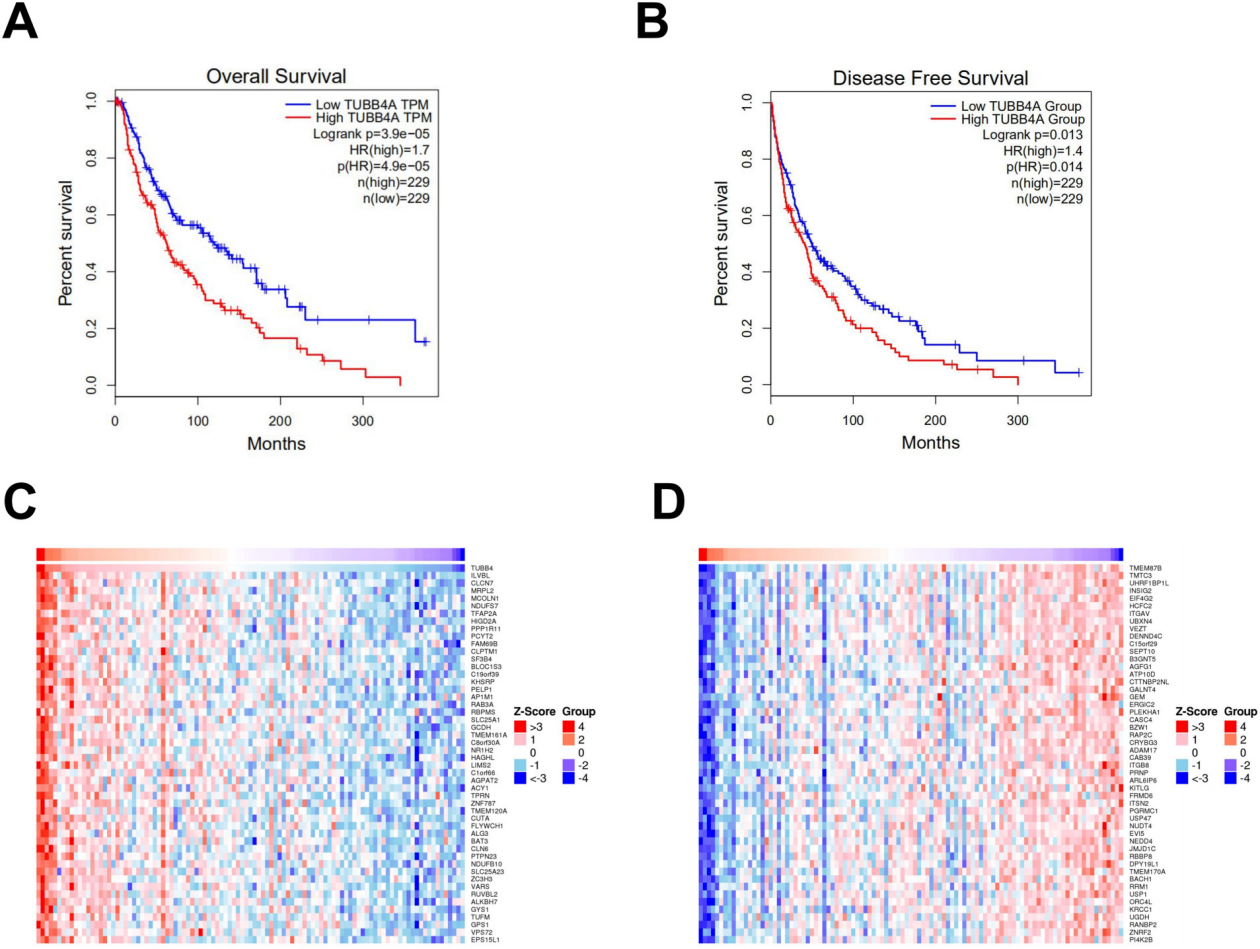
Survival and coexpression analyses. (A, B) OS and DFS survival curves of TUBB4A with significant log-rank *P*-values. (C, D) Heatmaps show the top 50 significant genes positively and negatively correlated with TUBB4A in SKCM. Red indicates positively correlated genes, and blue indicates negatively correlated genes.

Abbreviations: OS, overall survival; DFS, disease-free survival; SKCM, skin cutaneous melanoma.

The Kaplan–Meier survival analysis showed that patients with high TUBB4A expression had significantly shorter OS and DFS than those with low expression (Figure 3A and B). Then, the coexpression analysis of TUBB4A in SKCM cases was performed through LinkedOmics in TCGA database. As shown in Figure 3C and D, a total of 50 significantly coexpressed genes were demonstrated by a heatmap, either positively or negatively correlated with TUBB4A.

### Differences of TUBB4A Protein Levels Between Skin Cutaneous Melanoma and Normal Tissues

**Figure 4.**
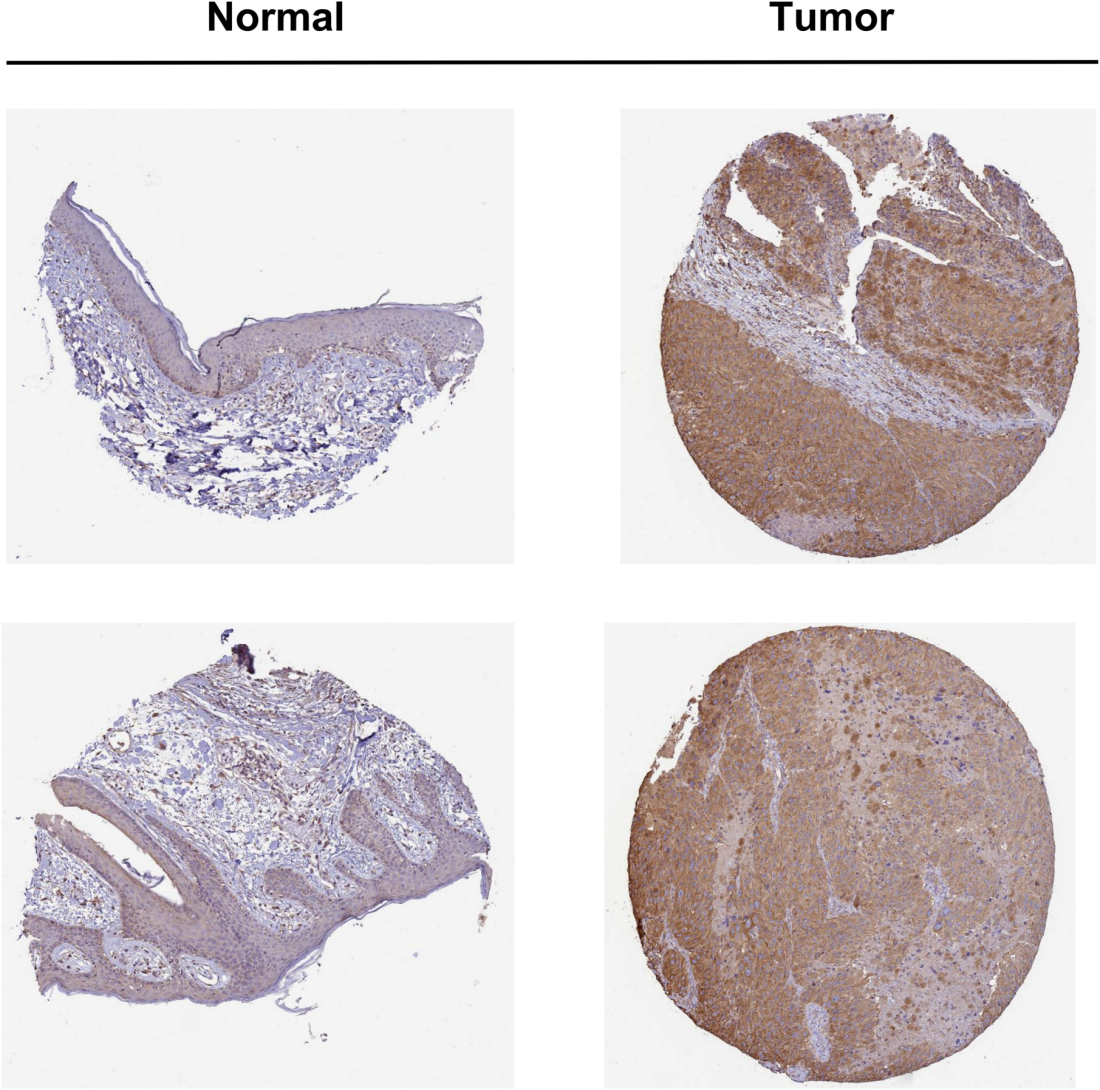
Immunohistochemical staining of TUBB4A in SKCM tissues and normal tissues in the HPA database. The protein level of TUBB4A expression was significantly higher in SKCM tissues than in normal skin tissues.

Abbreviations: SKCM, skin cutaneous melanoma; HPA, Human Protein Atlas.

Analysis of the HPA database showed that the immunohistochemical staining of TUBB4A was negative in normal tissues and positive in SKCM tissues (Figure 4). The protein level of TUBB4A expression was significantly higher in SKCM tissues than in normal skin tissues.

### TUBB4A Knockdown Significantly Inhibits Skin Cutaneous Melanoma Cell Proliferation

**Figure 5.**
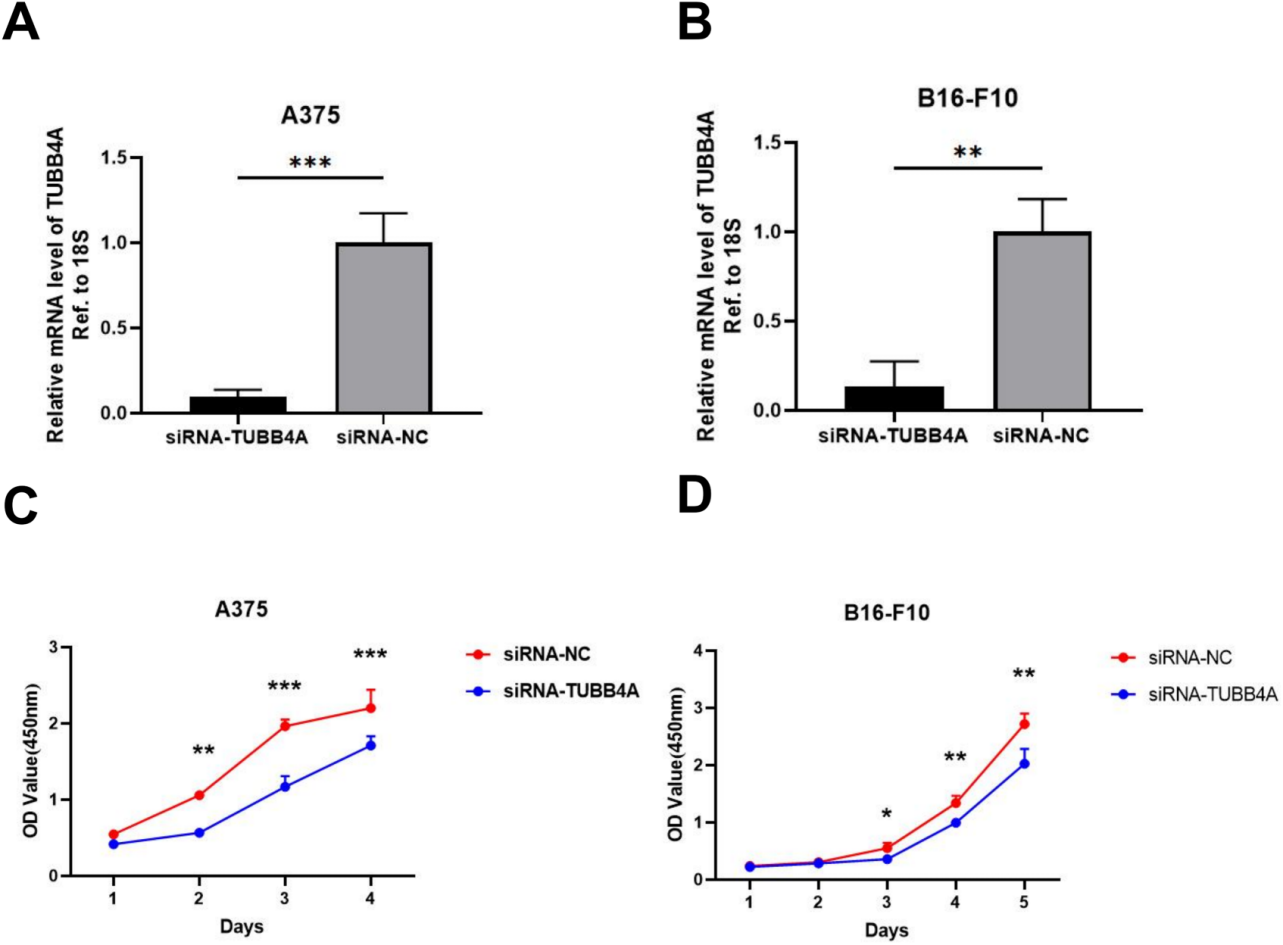
TUBB4A knockdown suppressed proliferation of SKCM cells. (A, B) Knockdown of TUBB4A was confirmed by qRT-PCR analysis in A375 and B16-F10 cells. (C, D) CCK8 assay of A375 and B16-F10 cells with or without TUBB4A knockdown. Data were represented as mean ± SEM of at least three independent experiments. *P < .05; **P < .01; ***P < .001. Data were analyzed using Student's *t*-test.

Abbreviations: SKCM, skin cutaneous melanoma; qRT-PCR, quantitative real-time PCR.

To further explore the role of TUBB4A in SKCM, A375 and B16-F10 cells were transfected with siRNA. The transfection sufficiently silenced TUBB4A expression. TUBB4A downregulation at the RNA level was evident at 48 h after transfection for both cell lines (Figure 5A and B). Using the CCK8 assay, cell viability was observed at 450 nm absorbance at 24, 48, 72, and 96 h. Both A375 and B16-F10 cells with TUBB4A knockdown showed reduced proliferation compared to that in the control group (siNC, Figure 5C and D). These results indicated that TUBB4A overexpression promoted the proliferation of melanoma cells.

### TUBB4A Knockdown Reduces Skin Cutaneous Melanoma Cell Migration *In Vitro*

**Figure 6.**
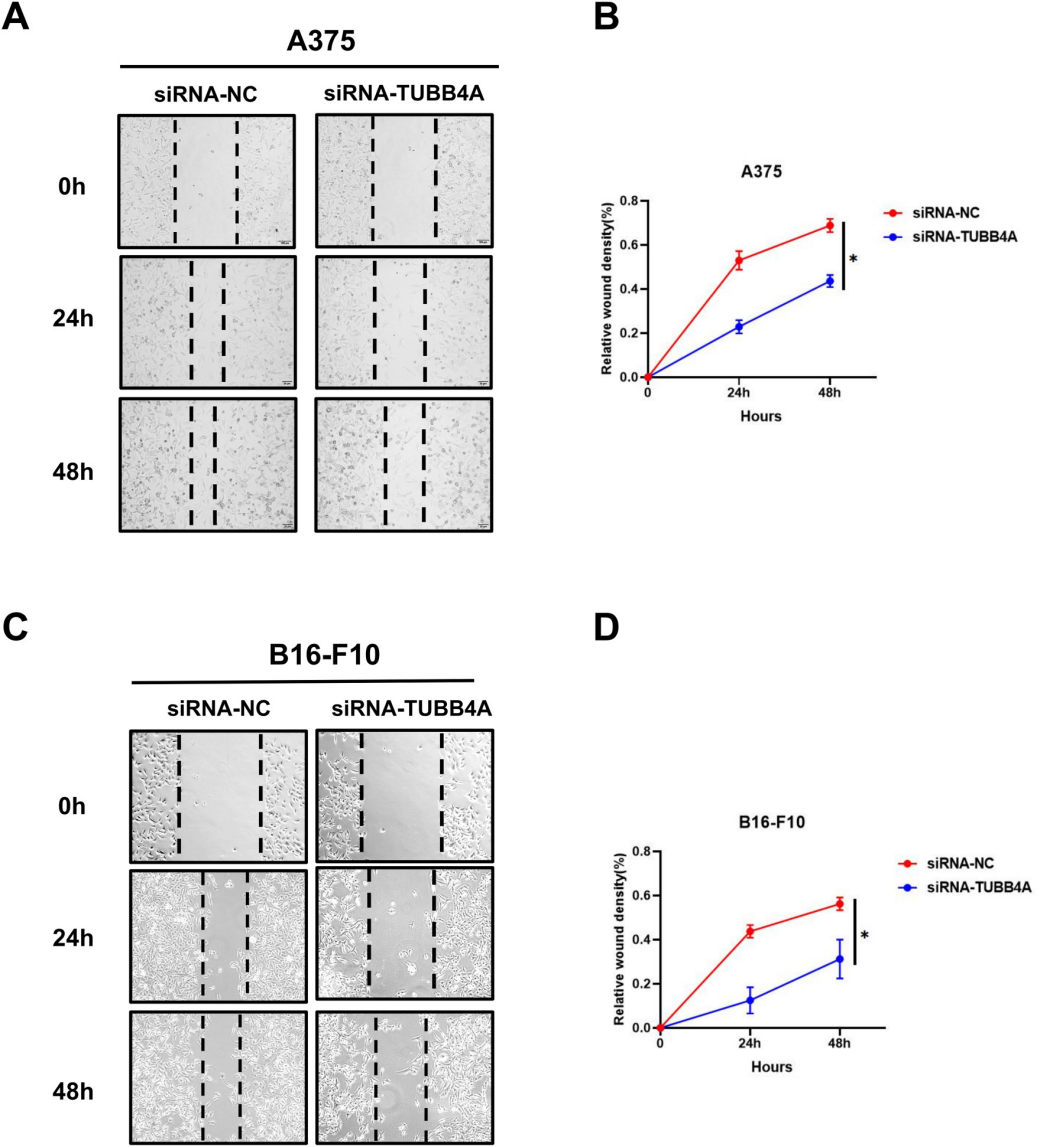
TUBB4A knockdown suppressed migration of SKCM cells. Scratch wound healing assay was applied in A375 (A, B) and B16-F10 (C, D) cells with or without TUBB4A knockdown. Data were represented as mean ± SEM of at least three independent experiments. *P < .05; **P < .01; ***P < .001. Data were analyzed using Student's *t*-test.

Abbreviation: SKCM, skin cutaneous melanoma.

SKCM cell migration capacity was detected by wound healing assay. A375 and B16-F10 cells, in which TUBB4A was knocked down by siRNA, showed a larger open wound area at 24 and 48 h, compared with that in the control group (Figure 6A and C). The difference in the open wound area at 24 and 48 h was quantified by calculating the percentage of change in the open wound area (Figure 6B and D, *P *< .05).

**Figure 7.**
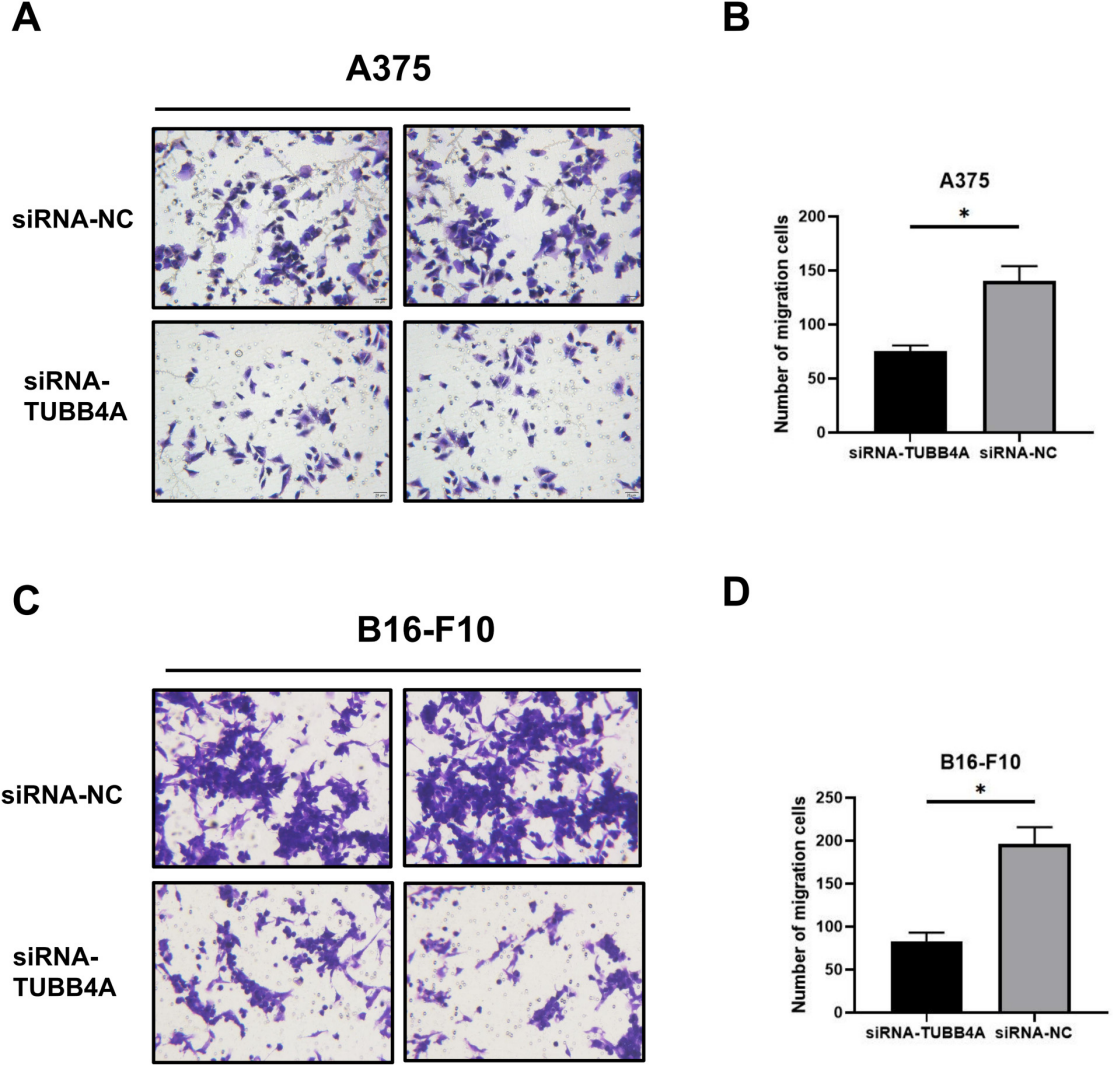
TUBB4A knockdown suppressed migration of SKCM cells by Transwell assay. Transwell assay was applied in A375 (A, B) and B16-F10 (C, D) cells with or without TUBB4A knockdown. Data were represented as mean ± SEM of at least three independent experiments. *P < .05; **P < .01; ***P < .001. Data were analyzed using Student's *t*-test.

Abbreviation: SKCM, skin cutaneous melanoma.

In addition, Transwell assay demonstrated that TUBB4A knockdown caused a strong reduction in migration of A375 cells (*t*-test, *P *< .05) and B16-F10 cells (*t*-test, *P *< .05) compared to that in the control group (Figure 7A to D). These findings indicated that TUBB4A overexpression could increase melanoma cell motility.

### TUBB4A Inhibitors Induce Apoptosis of Melanoma Cells

We further searched for small-molecule drugs that target TUBB4A in the DrugBank database. Five small-molecule agents potentially target TUBB4A. We selected two drugs, DHA and Nocodazole, the efficacy of which has been approved or is being trialed. DHA is an artemisinin derivative and antimalarial agent used in the treatment of uncomplicated *Plasmodium falciparum* infections and has been also reported to bind to TUBB4A protein.^36^ Nocodazole is a 16-membered macrolide that mimics the biological effects of taxol and functions as an inhibitor of microtubule function. Nocodazole has also been reported to inhibit the activity of TUBB4A.^37^

**Figure 8.**
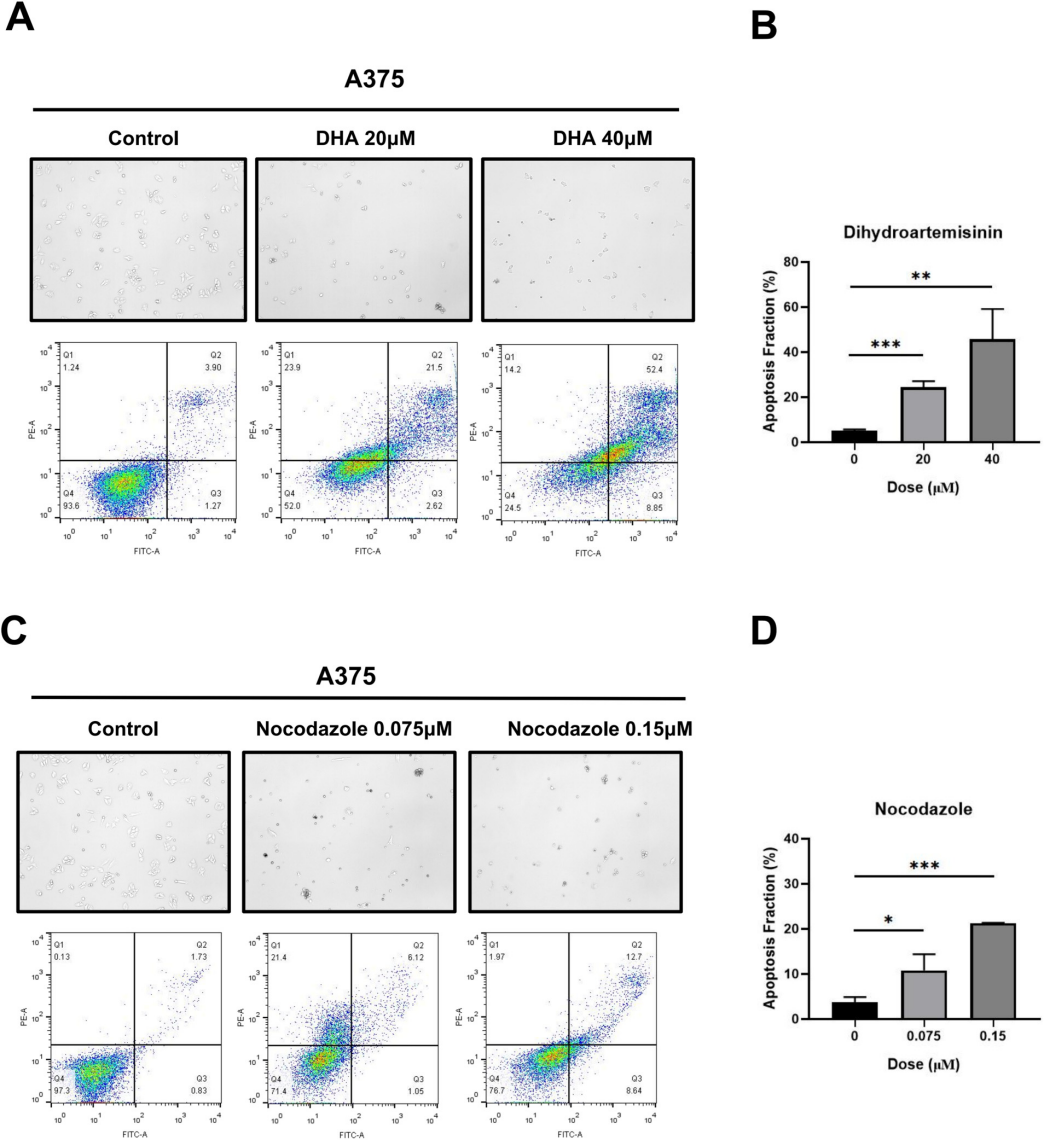
Dihydroartemisinin (DHA) and Nocodazole induced apoptosis of A375 cells. (A, B) Representative photograph of apoptotic A375 cells treated with or without DHA (20 or 40 μM). (C, D) Representative photograph of apoptotic A375 cells treated with or without Nocodazole (0.075 or 0.15 μM). Flow cytometry of apoptotic A375 cells by double staining with Annexin (An) V-FITC and PI. Quadrant Q1 represents An−/PI+ necrotic cells, Q2 represents An+/ PI+ late apoptotic cells, Q3 represents An−/PI viable cells, and Q4 represents An+/PI early apoptotic cells. Data were represented as mean ± SEM of at least three independent experiments. *P < .05; **P < .01; ***P < .001. Data were analyzed using Student's *t*-test.

We tested whether each drug can induce apoptosis of melanoma cells. A375 cells were treated with DHA (20 and 40 μM) for 48 h and Nocodazole (0.075 and 0.15 μM) for 24 h, separately. As shown in Figure 8A and C, microscopy revealed that exposure to either DHA or Nocodazole triggered fragmentation in the cells. A375 cell apoptosis induced by these two agents was confirmed by staining with Annexin V-FITC/PI and subsequent flow cytometry. As shown in Figure 8A to D, the proportion of apoptotic cells (quadrant Q2 An+/PI+ late apoptosis and quadrant Q4 An+/PI− early apoptosis) increased significantly in a concentration-dependent manner in cell lines after exposure to two drugs. Our results indicated the proapoptotic effects of DHA and Nocodazole in A375 cells.

### TUBB4A Inhibitors Modulate Melanoma Cell Cycle Progression

**Figure 9.**
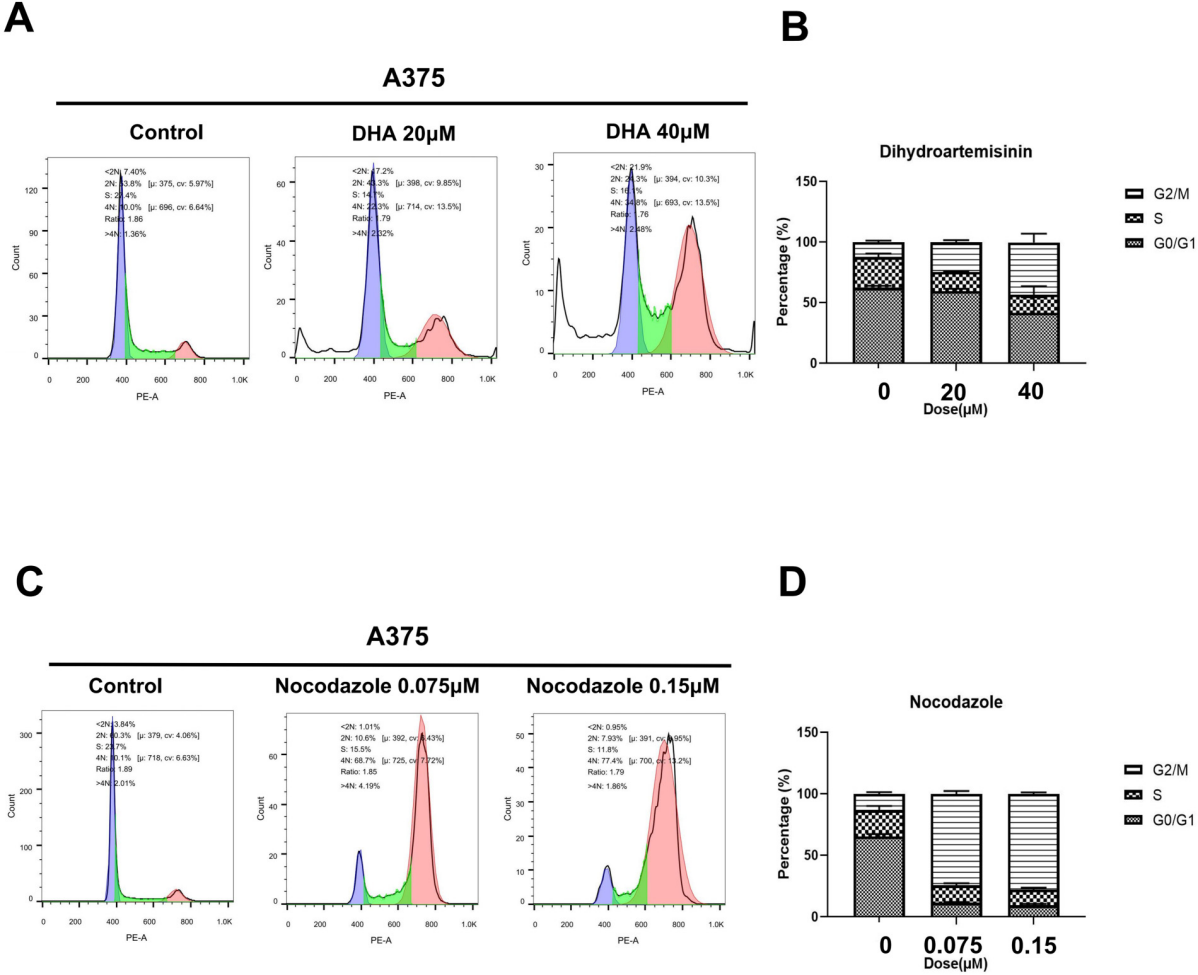
Dihydroartemisinin (DHA) and Nocodazole arrested the A375 cell cycle. (A, B) Cell cycle distribution of A375 cells treated with or without DHA (20 or 40 μM). (C, D) Cell cycle distribution of A375 cells treated with or without Nocodazole (0.075 or 0.15 μM). Both attached and floating cells were harvested for cell cycle analysis. Data were represented as mean ± SEM of at least three independent experiments. *P < .05; **P < .01; ***P < .001. Data were analyzed using Student's *t*-test.

We also examined the effect of two TUBB4A inhibitors on the A375 cell cycle using propidium iodide staining. A375 cells were treated with DHA for 48 h and Nocodazole for 24 h, respectively. With the increase in DHA concentration, the cell cycle of A375 cells (Figure 9A and B) was significantly blocked in the G2/M phase. As shown in Figure 9C and D, Nocodazole arrested the cell cycle, and the effects of 0.075 and 0.15 μM differed slightly. The results revealed that DHA and Nocodazole inhibited the proliferation of A375 cells by inducing cell cycle arrest.

## Discussion

In this study, we explored the prognostic value of TUBB4A for cutaneous melanoma and the potential efficacy of its inhibitors. We demonstrated that TUBB4A was highly expressed in SKCM, and its expression level was positively related to the viability of melanoma cells. TUBB4A knockdown by siRNA significantly inhibited the proliferation and metastasis of A375 and B16-F10 melanoma cells. Two TUBB4A inhibitors, DHA and Nocodazole, promoted melanoma cell apoptosis and induced cell cycle arrest in the G2/M phase *in vitro*.

A high level of class III beta-tubulin (β3-tubulin) is correlated with neoplastic cell hyperproliferation, metastasis, and resistance to chemotherapy.^38^ But few studies have delved into the role of class IV beta-tubulin (β4-tubulin) in tumor progression. TUBB4 and TUBB3 upregulation is coupled with increased migration of endothelial–mesenchymal transition–induced human microvascular endothelial cells (HMEC-1).^39^ Also, TUBB4 is necessary for the transport and localization of N-cadherin within the plasma membrane.^39^ Moreover, nonadherent culture induces paclitaxel resistance in H460 lung cancer cells via ERK-mediated upregulation of βIVa-tubulin.^21^ The above studies suggest that TUBB4A plays a crucial role in tumorigenesis. Here, we for the first time verified the role of TUBB4A in melanoma.

We retrieved four transcription factors (TFs) of TUBB4A from the KnockTF database,^40^ including HOXA1, IKZF2, TFAP2C, and TP53. Many studies have provided evidence that these TFs are closely coupled with TUBB4A in several carcinomas. For example, HOXA1 drives melanoma growth and metastasis via diverse cytokine pathways, including the TGFβ signaling axis. It also downregulates the expression of microphthalmia-associated TFs and other genes required for melanocyte differentiation.^41^ Maeda et al^42^ reported that the expression level of HOXA1 in melanoma with distant metastasis is higher than that in unmetastasized melanoma. MicroRNA-214 suppresses TFAP2C to propel melanoma progression.^43^ The levels of TFAP2C methylation are evidently different between primary lesions and metastases of melanoma.^44^ Tubulin acetylation favors the recruitment of molecular chaperone Hsp90 to microtubules and the binding and signaling of kinase Akt/PKB and TF p53.^45^ Furthermore, Arai et al^46^ reported that tubulin inhibitor *vinca alkaloid* hijacks tumor suppressor p53 protein to enhance the expression of class II beta-tubulin isotype (mTUBB2) in mouse B16-F10 melanoma cells. Other studies found that p53 transcriptionally downregulates microtubule-associated protein 4^47^ and that human breast carcinoma cells with a mutated p53 gene display an increased level of class IV β-tubulin.^48,49^

We also investigated the downstream factors of TUBB4. Proximity ligation assay (PLA) and immunoprecipitation studies confirm that GLUT1 interacts with TUBB4 in human glioblastoma specimens. Treatment of glioblastoma cells with a TUBB4 inhibitor, *CR-42-24*, reduces the expression of GLUT1; however, TUBB4 expression is unaltered upon treatment with GLUT1 inhibitor fasentin.^18^ These indicate that GLUT1 may be a downstream gene of TUBB4A. Also, ITSN1-L, one isoform of Intersectin1, can strengthen cell–cell adhesion by upregulating N-cadherin expression and facilitating relocalization to the membrane by ANXA2 and TUBB3/TUBB4.^50^

It has been reported by scholars that ERK signaling mediates the upregulation of TUBB4A and confers cancer cells with resistance against paclitaxel. Moreover, CR-42-24, as a TUBB4 inhibitor, reduces the expression of GLUT1 in glioblastoma cells. A mechanistic study suggested that mutual regulation between GLUT1 and the MEK/ERK cascade relays communication between glucose uptake and stress response.^51^ We consider that TUBB4A may participate in the constitutive activation of the MEK/ERK pathway and further cooperates with GLUT1 to affect BPs, such as glucose uptake. Through the TGFβ signaling axis and other cytokine pathways, HOXA1 may regulate TUBB4A to drive melanoma growth and metastasis, which should be proven by experimental studies in the future.

The main limitations of our study should be acknowledged. We provide a literature review and bioinformatics analysis related to TUBB4A, but they still lack wet-lab experiments to reveal the related cellular mechanism. Despite this limitation, our findings demonstrated the biological function of TUBB4A in SKCM for the first time, suggesting it is a promising prognostic marker and therapeutic target.

## Conclusion

TUBB4A functions as a driver in increasing melanoma cell proliferation and motility, and TUBB4A inhibitor agents can significantly induce melanoma cell apoptosis and G2/M cell cycle arrest. TUBB4A may be a potential prognostic marker and therapeutic target of melanoma.

## Abbreviations

α-MSH: α-melanocyte-stimulating hormone
BPs: biological processes
CCs: cellular compositions
DEGs: differentially expressed genes
DFS: disease-free survival
DHA: Dihydroartemisinin
DMEM: Dulbecco's modified Eagle's medium
FBS: Fetal bovine serum
GEO: Gene Expression Omnibus
GO: Gene Ontology
GTEx: Genotype-Tissue Expression
HPA: Human Protein Atlas
KEGG: Kyoto Encyclopedia of Genes and Genomes
MFs: molecular functions
OS: overall survival
qRT-PCR: quantitative real-time PCR
SKCM: skin cutaneous melanoma
TCGA: The Cancer Genome Atlas
TUBB4A: β-tubulin 4A
